# Exploring motherhood dilemmas and coping strategies among mothers of children with autism: a qualitative study in mainland China

**DOI:** 10.3389/fpsyt.2025.1569928

**Published:** 2025-06-09

**Authors:** Jun Zhou, Yuanhong Ning

**Affiliations:** Department of Social Work, School of Philosophy and Social Development, Shandong University, Jinan, Shandong, China

**Keywords:** mothers of autistic children, motherhood dilemmas, coping strategies, maternal resilience, qualitative research

## Abstract

**Background:**

Under the traditional division of gender roles in China, mothers often shoulder more responsibilities for caring for children with autism. This article is the first attempt to explore the motherhood practices of mothers of autistic children.

**Method:**

Through in-depth interviews with eight mothers, revealing three major themes: (1) the mothering dilemmas encountered by mothers of children with autism, (2) why mothers of autistic children choose to stay the course, and (3) the maternal adjustment practices of mothers of children with autism.

**Results:**

This study uncovers the challenges encountered during the nascent phases of these mothers’ parenting journeys. However, these mothers showed remarkable resilience and adaptability to these challenges. They also developed various motherhood adjustment strategies to cope with these dilemmas.

**Conclusions:**

The findings emphasize the maternal resilience demonstrated by Chinese mothers of children with autism in the parenting process and the sources of this resilience. They provide new perspectives for understanding this group’s parenting practices, informing related policy development, and supporting service provision.

## 1 Introduction

Autism is a complex neurodevelopmental disorder characterized by early onset and lifelong persistence (1). In China, approximately 160,000 new cases are diagnosed annually, with the total number exceeding 2 million (2). Individuals with autism often face challenges in emotional regulation and exhibit internalizing symptoms such as anxiety and depression, increasing the caregiving burden (3). Despite gradual improvements in China’s social welfare system for families with disabilities (4), support services specifically targeting families with autistic children remain limited (5). Besides, societal factors, including maternal emotional expectations, gender norms, privatization of domestic services, insufficient public care resources, and male preference in the public sphere, collectively position mothers as primary caregivers (6). Consequently, caregiving responsibilities for children with autism predominantly fall on mothers.

Research shows that mothers of children with autism are far less likely to abandon their children than fathers. Motherhood is often viewed as instinctual, and from the initial suspicion to the diagnosis of autism, mothers typically face this journey alone (7). Throughout this process, they endure economic, social, and caregiving-related pressures. Many report that raising a child with autism has profoundly impacted their lives, disrupting family routines, hindering careers, and isolating them from mainstream society (8). In China, studies have noted that these mothers often experience dysregulated parent-child interactions and deep parental distress (9). The strain may extend to marital and family conflicts, with mothers frequently becoming sole caregivers, leading to acute loneliness (10).

Nevertheless, caregiving is not entirely negative. Some mothers believe that raising an autistic child has broadened their perspectives, deepened their understanding of others, and strengthened their sense of life’s meaning (11). Others have redefined their roles, balancing work and caregiving, adjusting expectations, gaining social identity, and becoming community advocates (12). Positive maternal cognition is strongly linked to parenting outcomes; those who see their children progress tend to gain a stronger sense of efficacy (13). Despite extensive attention to the challenges faced by these mothers, few studies explore how they cope. While some research touches on positive experiences, it rarely investigates the sources of these transformations or systematically analyses the coping strategies involved. Moreover, the specific mothering experiences of Chinese mothers remain under-researched.

The study of motherhood originated in Western feminist scholarship during the 1960s–1980s (14), evolving into two key concepts: motherhood and mothering. Motherhood refers to the social and cultural norms that define women’s roles as mothers, how and why women “should” mother. In contrast, mothering emphasizes women’s lived experiences and subjective practices of being mothers (15). Hayes (16) introduced the concept of intensive motherhood, describing a child-centered, emotionally and physically demanding ideology in which mothers bear primary responsibility for their children’s development. In this model, children’s needs are prioritized, often at the expense of mothers’ well-being (16). As diverse forms of motherhood have emerged, the challenges of mothering have also become more visible. The notion of the motherhood penalty captures the systemic discrimination women face due to reproductive and caregiving responsibilities (17, 18). Responding to the dominance of intensive motherhood, Elliott et al. (19) proposed extended motherhood, wherein low-income mothers, constrained by work and limited resources, rely on extended kin or neighbors for childcare. O’Reilly (20) further advanced the idea of maternal empowerment, arguing that motherhood can be a female-defined, liberatory experience. Through conscious resistance to patriarchal norms, mothers may reclaim agency and redefine their roles on their terms.

Motherhood studies examine women’s roles and responsibilities as mothers and the social expectations and evaluations surrounding these roles. As a feminist theoretical framework, this field adopts a woman-centered perspective, situating child-rearing within its socio-cultural context. It critically reflects on the institutional and cultural foundations that shape women’s caregiving responsibilities, offering new insights into the parenting experiences of mothers of children with autism. Based on this perspective, this study adopts a qualitative approach to explore how these mothers navigate their caregiving roles, balancing societal expectations with personal needs and developing coping strategies. In-depth interviews were conducted with eight mothers of autistic children, guided by the following research questions:

- What are the parenting experiences of these mothers after their child is diagnosed with autism, and what challenges do they face?
- Why do mothers of children with autism choose to persevere in the face of challenging parenting practices?
- What maternal coping strategies have they developed during this process?

## 2 Methods

### 2.1 Research design and participants

This study employs qualitative research methods, utilizing in-depth interviews to explore the subjective experiences of mothers raising children with autism. We use purposive sampling to select participants who meet the inclusion criteria, ensuring they have the relevant experiences and knowledge to address the research questions. All participants were recruited through Organization C, an autism parent support center that provides public welfare services, supportive training, and psychological counselling for families of individuals with autism. Families must submit formal documentation issued by a tertiary (Grade III-A) hospital in China to receive services from Organization C. This documentation typically includes a confirmed diagnosis of autism spectrum disorder (ASD), based on a variety of tools, such as the Autism Diagnostic Observation Schedule, Second Edition (ADOS-2) or the Childhood Autism Rating Scale (CARS). Therefore, before participating in this study, each mother had already obtained medical documentation confirming her child’s diagnosis and severity level.

The inclusion criteria for participants were: (a)mothers whose children had received a formal clinical diagnosis of ASD by a qualified medical institution in mainland China; (b) mothers who were the primary caregivers of children diagnosed with autism spectrum disorder (ASD) for more than five years; and (c) mothers who were able and willing to share their caregiving experiences through in-depth interviews. The exclusion criteria included: (a) fathers or other family members acted as caregivers, so mothers were not the primary caregivers; (b) mothers whose children were diagnosed with other developmental disorders without a formal ASD diagnosis.

Eight mothers of children with autism were included in the study, ranging in age from 35 to 55 years. All participants were full-time mothers with a clear division of family roles, where fathers were responsible for financial support, and mothers handled domestic and caregiving duties. The children’s ages ranged from 8 to 22 years, with five diagnosed with moderate to severe autism and two with mild autism. These mothers possessed extensive caregiving experience and demonstrated significant resilience, with notable shifts in their attitudes from the early to later stages of their children’s development. One participant even established a social work organization dedicated to supporting mothers of children with autism, highlighting the depth of their experiences. Demographic information, including the mothers’ educational backgrounds and the children’s ages at diagnosis, is presented in **Table 1**.

**Table 1:** Participant information.

Participant Number	Years of Full-Time Parenting	Education Level	Number of Children in the Family	Age of Autistic Child (Years)	Level of Severity
Case1	10yrs	Bachelor’s	1	13yrs	Moderate to Severe Autism
Case2	8yrs	Bachelor’s	1	11yrs	Moderate to Severe Autism
Case3	12yrs	Junior High	1	15yrs	Moderate to Severe Autism
Case4	18yrs	Junior High	1	22yrs	Mild Autism
Case5	16yrs	Junior High	2	20yrs	Mild Autism
Case6	5yrs	Bachelor’s	2	8yrs	Moderate to Severe Autism
Case7	12yrs	Associate’s	2	14yrs	Moderate to Severe Autism
Case8	17yrs	Junior High	1	19yrs	Moderate to Severe Autism

### 2.2 Data collection

This study employs in-depth interviews as the primary method of data collection. The objective of the research is to explore the mothering experiences of mothers of children with autism in China, with a focus on the core theme of “mothering experiences”, which encompasses the mothers’ narratives and subjective feelings, which can be divided into two main aspects: the challenges encountered in raising children with autism and their coping strategies. Regarding the challenges of mothering, the mothers were asked questions such as “How did you feel after your child was diagnosed with autism?” and “What difficulties have you encountered in the process of raising your child?” Regarding personal coping, questions included “How did you address these difficulties?” and “What has kept you committed to parenting practices without giving up?” Through these profound conversations, we aim to gain an in-depth understanding of the experiences and coping mechanisms of mothers in China who are raising children with autism.

Each participant was interviewed for 1–2 hours, with all in-person interviews conducted at Organization C. With explicit informed consent from participants, all interviews were audio-recorded. During the interviews, the researchers followed a semi-structured interview guide and posed follow-up questions based on the flow of the conversation to elicit more in-depth information. In addition to verbal responses, the researchers also documented participants’ body language and emotional reactions. Each of the eight participants was interviewed multiple times. No new codes or key information emerged during the final three interviews, indicating that data saturation had been reached.

### 2.3 Data analysis

Thematic analysis was employed to analyze the data, following the approach outlined by Braun and Clarke (21). This method involves identifying key themes and patterns through repeated reading and coding, uncovering underlying structures and relationships within the data to understand the core experiences and feelings of the research participants. The analysis process began with familiarization with the data, which involved repeated reading of the interview transcripts to gain a deep understanding of the participants’ experiences. Next, initial coding was conducted, where meaningful data segments were labelled with descriptive codes, such as “limited involvement of husbands in the parenting process.” Subsequently, potential themes were identified by grouping related codes. Several core themes emerged, including social challenges such as “difficulties in parenting,” “stigma by association,” and “insufficient social support,” as well as personal-level difficulties like “feelings of loneliness in the parenting process.” Positive coping strategies, such as “seeking support and resource integration,” were also identified. These initial themes were reviewed and refined to ensure logical and theoretical consistency. Through iterative discussions and reflections, the definitions and names of the themes were finalized, resulting in the core themes of this study.

### 2.4 Rigor

This study is an exploratory qualitative inquiry employing semi-structured in-depth interviews. The research report was prepared by the Consolidated Criteria for Reporting Qualitative Research (COREQ) (22). Multiple strategies were employed to ensure the credibility of the study. First, prior to formal data collection, the semi-structured interview guide was developed based on a systematic review of relevant literature. Second, all researchers had prior experience conducting interviews with mothers of children with autism and received training in qualitative research methodology before commencing the study. Two researchers independently analyzed the data. Regular team meetings were held to discuss and resolve discrepancies in coding, categorization, and identifying and naming subthemes and overarching themes. Furthermore, we detailed the research context, study design, participants, and data collection and analysis procedures. An audit trail and reflective journals were maintained to examine the researchers’ perspectives and preconceptions, ensuring the findings were firmly grounded in the data.

### 2.5 Ethical considerations

The study adhered strictly to ethical principles, and the institutional ethics committee approved the research protocol. All participants provided informed consent, and they were provided with detailed information about the study’s purpose, procedures, potential risks, and benefits. All identifiable information was removed to ensure confidentiality.

## 3 Results

In this section, we present the core findings of the study, which primarily focus on exploring the mothering experiences of mothers of children with autism. The research reveals that the mothering experiences of mothers of children with autism can be delved into the following aspects: (a) the mothering dilemmas faced by mothers of children with autism; (b) why mothers of children with autism choose to persevere; (c) the mothering adaptation strategies developed by mothers of children with autism in the process of perseverance.

### 3.1 The mothering dilemmas encountered by mothers of children with autism

Interviewees generally expressed that they encountered numerous challenges in raising children with autism. In the following sections, we will delve into the specific difficulties faced by mothers of children with autism during this process, including the imbalance in time allocation and the disruption of parent-child interaction, the absence of paternal roles and the imbalance of childcare responsibilities, as well as the accompanying stigmatization and lack of support systems.

#### 3.1.1 Imbalance in time allocation and disruption in parent-child interaction

Traditional patriarchal norms continue to shape gender roles in contemporary Chinese families. Mothers, often positioned as primary caregivers due to both gender ideology and the economic division of labor, face the brunt of childcare responsibilities, especially after their child is diagnosed with autism. Many choose to quit their jobs and become full-time caregivers, sacrificing personal space and social engagement, and feeling physical and emotional exhaustion. Case 3 described a complete shift in her life trajectory:

I took him to rehab at just over 2 years old. There was no rehab at 10 years old. Before that, it had been half a day at a special school and half at the rehab center. I took him daily; I got up at 5:00 a.m., got up early, and was tired. (case 3)

In addition, Children with autism often have deficits in emotional communication and social motivation, which hinder mutual interaction and emotional exchange with their mothers. Such dysfunction frequently leads to frustration and emotional fatigue among mothers (23), especially when accompanied by aggressive behaviors. Case 1 described how her child, after a decline in social functioning, developed seasonal episodes of violence, including yelling and hitting, prompting the parents to wear helmets for protection. Under the influence of traditional Chinese family values, these mothers experienced a strong sense of loss in the early stages of parenting. They felt pessimistic and despairing about their children’s future and the prospects of their families.

I hope I die after him…… Parental enthusiasm and passion just get drained by these things. Sometimes, it is almost like you do not want to live; you do not find it interesting. (case 1)

#### 3.1.2 Lack of paternity and imbalance of parenting responsibilities

Due to limited social assistance and the high costs of autism interventions, families often experience intense financial strain. It typically leads to a gendered division of labor, where mothers become full-time caregivers while fathers focus on earning an income. Under such stress, some fathers disengage from parenting altogether, even using work as a pretext to avoid caregiving responsibilities (12). In Case 1, the husband worked abroad after their child’s diagnosis, leaving the mother to parent alone for over a decade. Even after returning, he rarely participated in parenting.

*My husband left the country as soon as the child found out about autism at age 2,……. There’s nothing to talk about; it is a pattern: hubby earns the money, and then mom carries it. The husband’s support is very little…… Just don’t give me any more trouble—that’s all I ask……I am taking a related intervention course and pressing him to look at it, but he won’t look at it.* (case 1)

Similarly, in Case 2, the mother resigned to provide full-time care, and her husband bore financial responsibilities. However, her husband rarely gave her emotional support.

*I was crying on the phone, telling him how hard it was, but he just said: ‘I gave you money. What else do you want? Don’t give me negative vibes.’ That still hurts.*(case 2)

In modern Chinese society, gender norms, service privatization, and male privilege in the public sphere reinforce caregiving as a maternal duty (6). Fathers often assume only the role of financial providers, remaining detached from their children’s education and their partners’ emotional needs. However, autism care demands systematic, sustained engagement. This emotional distance makes mothers feel isolated and unsupported in parenting and marriage. As Case 1 said, after years of autism care, she and her husband no longer seemed to have the emotional connection that usually exists in a marital relationship, but more of a “living together” partnership. And these mothers have higher levels of stress and psychological frustration than fathers (24).

#### 3.1.3 Associated stigma and lack of social support

When discussing external challenges in parenting, interviewees commonly expressed feelings of isolation and stigmatization as well as the lack of supportive environments. According to Foucault’s biopolitical analysis, modern society has turned health into an individual moral obligation (25, 26). This shift moralizes medical discourse and devalues those who deviate from the norm, including children with autism. As a result, their mothers are perceived as caregivers and morally accountable for their child’s condition (27). In Chinese culture, Confucian values amplify this stigma by framing motherhood as a site of honor and duty. Mothers of autistic children are often blamed for giving birth to “flawed” offspring, with the child’s diagnosis attributed to maternal negligence during pregnancy or infancy (28). They are also expected to be “good mothers” responsible for their children’s development and social outcomes. Deviations from these expectations are often met with blame and judgment (29).

When you take the child out, a lot of people aren’t really fond of it….We basically don’t have a common language with the normal parents in the society, we can’t talk about anything with them. (case 4)

Failure to meet caregiving expectations may lead to social ostracism. As Ryan and Runswick-Cole (30) noted, poor child management is seen as a sign of maternal irresponsibility, provoking stigma from both community and family members. Case 1 recalled questioning herself after the diagnosis, worrying about prenatal care or genetic issues, yet test results showed no medical explanation. She quit her job and sought rehabilitation services across cities, but when these efforts showed little progress, she returned home, only to face familial disappointment:

I quit my job and took my child to various places for rehabilitation, but it was hardly effective either, so I finally had to take my child home, and my family felt that I went out and spent so much money and my child didn’t get much better, and they were very dissatisfied with me anyway. (case 1)

At present, China has yet to establish a comprehensive care system for autistic children. Most mothers are heavily involved in their children’s rehabilitation and often find themselves “going it alone” in parenting, making it challenging to build a robust social support network independently. Although a few rehabilitation institutions exist, their limited specialization leads many mothers to forgo long-term institutional care and instead rely on family-based rehabilitation and intervention. As Case 1 illustrated, due to inadequate social support and limited access to information during early intervention stages, mothers frequently missed the critical window for autism intervention. It is often because they are unaware of relevant policies and benefits and cannot mobilize necessary resources for their children. Additionally, influenced by traditional beliefs, some mothers feel embarrassed to expose their children publicly or fear that acknowledging their children’s disabilities might restrict future opportunities. So they may conceal their children’s conditions and forgo applying for disability certificates, thereby losing access to available social support.

### 3.2 Why mothers of autistic children choose to stay the course

When asked how they persevered through the challenges of parenting, these mothers revealed multiple psychological dimensions of struggle. The following sections will explore the reasons behind their perseverance, including (a) the return of fatalism under the traditional medical discourse, (b) the internalization of responsibility under the norms of moralized motherhood, and (c) the formation of neurodiverse perspectives under the construction of pluralistic knowledge systems.

#### 3.2.1 The return of fatalism under the traditional medical discourse

Local ethical systems can profoundly influence patients and caregivers (31). Fatalism, a long-standing worldview and ideology, posits that unchangeable determinants underlie many mysterious and fateful phenomena experienced by humans. Fatalism is often a robust explanatory framework deeply embedded in Chinese culture, especially during sudden changes or severe crises (32). In the early stages following diagnosis, mothers commonly undergo a process of helpless acceptance. Confronted with the diagnosis and the limitations of traditional medical discourse, many mothers unconsciously turn to traditional Chinese fatalism, “resigning to their fate” by accepting their children’s autism. As Case 3 describes, “It is something that we are subjected to; when we first started, we did not recognize it. We thought it was such bad luck to have this child. Then we felt we had to recognize it after a long time.” This acceptance represents a “self-recognition of bad luck” within traditional medical discourse, a helpless submission to unfortunate fate. Through this interpretation, mothers find support and comfort, achieving psychological self-compatibility (33). It also provides an essential source for them to find positive strength (34).

#### 3.2.2 The internalization of responsibility under the norms of moralized motherhood

In Chinese society, ethical norms govern the gender division of labor within families. Mothers are judged based on their ability to raise children who conform to societal expectations, and having a child with developmental differences often exposes them to moral scrutiny from the wider community. This ideology shapes the values of mothers of children with autism, who, particularly in the early stages following diagnosis, may experience stigmatization related to their child’s developmental differences and feelings of marginalization (35). Compared to fathers, they bear heavier moral burdens and often feel guilt or self-blame if they perceive themselves as failing to meet society’s ideals of motherhood (36, 37). Furthermore, illness is intricately connected to broader family, social, and interpersonal relationships, especially intimate family bonds (31). As the primary caregiver and principal planner of their autistic child’s education, mothers are often held accountable for their child’s successes and failures. This societal expectation profoundly impacts mothers of autistic children, who internalize and construct a sense of moral obligation to assume responsibility for caring for and addressing their children’s challenges, becoming the central figures of caregiving responsibility (38). This moralized motherhood norm profoundly influences many mothers of autistic children. Several interviewees expressed sentiments such as, “If mom won’t take care of them, who will?” They have internalized this norm, channeling it into intensive parenting, with a sense of responsibility as a crucial motivation for their perseverance.

#### 3.2.3 The formation of neurodiverse perspectives under the construction of pluralistic knowledge systems

Explanatory disease models encompass lay theories of illness and patient-centered explanatory models, primarily derived from patients’ observations and experiences, as well as expert biomedical and lay medical frameworks (31). We found that some mothers developed a neurodiversity perspective on parenting. Neurodiversity, a term inspired by biodiversity, posits that neurological differences, such as those seen in autistic individuals, are natural variations within human diversity. It asserts that variations in brain structure are as common as other human traits, including race, gender, culture, and sexual orientation (39). These mothers view certain stereotypical behaviors of their children not as misbehavior, but as characteristic expressions of autism. Through their lived experiences and observations, they formulated a distinctive understanding and explanatory model of autism that contrasts with traditional pathological frameworks, emphasizing the individual’s and their family’s perspectives (40). For instance, Case 8 emphasized repeatedly that autism is merely one trait of the child, and the label should not restrict their development. Embracing this neurodiverse perspective enabled mothers to perceive positive parenting as a source of hope, recognize the value and benefits of motherhood throughout the parenting journey, and identify effective coping strategies.

### 3.3 Coping strategies: maternal adjustment practices for mothers of children with autism

Most interviewees said that after accepting that their children were sick, in the course of parenting, they would develop a series of motherhood adjustment strategies to redefine their role as mothers and relieve their parenting stress and dilemmas as their parenting practice progressed. In the following chapters, we will explore (a) individual capacity building and generation of personalized parenting strategies; (b) negotiation of family responsibilities, promoting paternal involvement in parenting; and (c) social support reconstruction, reproducing values in the mother’s community.

#### 3.3.1 Individual capacity building: generation of personalized parenting strategies

In the course of raising children with autism, many mothers gradually develop personalized strategies to cope with parenting stress and redefine their maternal identity. This process involves acquiring medical knowledge and integrating insights from psychology, education, and social work to build a holistic, child-centered approach to care. Case 1 said that she focused exclusively on her child’s rehabilitation initially, seeking improvement through medical intervention. However, she soon realized that many rehabilitation centers lacked professionalism and failed to meet the diverse needs of autistic children. Over time, she recognized that effective parenting required more than standardized training, it demanded an in-depth understanding of her child’s unique emotional and behavioral patterns. It prompted her to pursue formal education in social work and related fields. She eventually obtained a social worker’s license and founded a nonprofit organization supporting other mothers of autistic children. Her approach, shaped by interdisciplinary learning, enabled her to develop tailored caregiving strategies that eased her parenting burden and significantly improved her child’s communication and self-care abilities.

The first stage was rehabilitation, from when my child was two until he was nine, I focused on medical treatment. However, I found that it didn’t work. The system was fragmented, with no integration between medical care, education, or understanding of the child’s characteristics. So I took the initiative to integrate them myself. Social work helped me accept my child, because it shifted how I viewed him. (Case 1)

Similarly, other mothers interviewed described how long-term interactions with their children allowed them to observe behavioral cues and emotional responses, gradually shaping adaptive and responsive parenting methods. These mothers emphasized that observing patterns, reflecting on behaviors, and adjusting accordingly helped them feel more competent and emotionally resilient.

I understand my child very well. Sometimes I can predict what he’s going to do next just by a subtle gesture or expression. Then I can respond by preventing or guiding him through a meltdown. But first, I always try to figure out why he’s doing it. (Case 5)

Building parenting capacity enhances mothers’ caregiving effectiveness and improves their emotional well-being. Several mothers shared that witnessing their children’s progress and receiving even subtle forms of positive feedback gave them a renewed sense of maternal value and identity, transforming motherhood from a site of stress to one of growth.

#### 3.3.2 Negotiating family responsibility: promoting paternal involvement in parenting

Although mothers remain the primary caregivers in most families, many do not passively conform to traditional norms of motherhood (41). Instead, they adopt strategic negotiation to encourage paternal involvement in child-rearing, aiming to redistribute caregiving responsibilities within the family structure. Case 1 consciously involved her husband in childcare after he returned to China, coordinating her working hours with her husband and handing over the responsibility of caring for her children during her working hours. The involvement in childcare positively changed her husband’s role and mindset, and he attempted to be more open about taking the children out in public.

Now that I’m not like I used to be, I also need a break and I have my own things to do, so I now reserve my Monday through Friday time for my child, and leave him with his dad for the other afternoons to bring him up. (In fact) he is now slowly getting involved and is able to bring it up well. (case 1)

Case 7 also realized the importance of her husband’s involvement in children’s upbringing, especially in skill development and sex education. She acknowledged the father’s contribution to her children’s development, noting that through this involvement, the father received positive feedback from his children, which led to a greater willingness to be involved in parenting.

Learning to ride a bike was thanks to his dad—he held on from behind and let go at just the right moment. That gave him a special sense of pride. And every year, his dad plans a family trip and makes sure to book his favorite window seat … For sex education, we leave it to his dad, it’s more appropriate since he’s a boy. They’re very close. (Case 7)

When mothers consciously encourage and enable fathers to participate in childcare, fathers become active contributors to caregiving. It represents a redefinition and rebalancing of traditional gender roles and labor divisions within the family by mothers, reflecting their positive efforts to challenge conventional motherhood ideologies, assert their agency, and deconstruct dominant motherhood discourses. Strategically negotiating family responsibilities to promote father involvement alleviates mothers’ caregiving burdens, strengthens the father-child bond, and supports the child’s overall development. For mothers with active paternal involvement, previously weakened family support networks are re-established, providing emotional support that indirectly enhances the mother-child relationship and positively influences mothers’ parenting behaviors and experiences (42).

#### 3.3.3 Social support reconstruction: reproducing values in mothers’ communities

As mothers embrace their caregiving roles and develop coping skills, they strive to overcome structural and cultural barriers, reconstruct social support networks (43), and plan for their children’s futures despite limited resources. Case 1 shared that after achieving some success in caring for her child, she became acutely aware of the helplessness faced by this group and, together with several other mothers, attempted to form an organization of parents of autistic children for mutual support. She said, “My child missed out on the golden age of development, and I have taken many detours, but I do not want more parents to make the same mistakes.” As more parents joined, the organization expanded beyond a small social circle into a support center where mothers provide emotional companionship, share experiences, and recommend resources, thus building an increasingly robust support network.

Although limited, this social support is vital for mothers’ parenting journey. By creating and sustaining new networks, mothers better adapt to their caregiving roles and alleviate the psychological stress of parenting. The support system offers emotional comfort, helps mothers find joy in parenting, and maintains a positive attitude amid challenges. Within these networks, mothers acquire new perspectives and strategies to cope more effectively with parenting difficulties and plan for various situations. This growing support network also attracts more mothers to participate, fostering a sense of strength and self-worth.

I used to not be a patient person with a quick temper. It is my child who has made me what I am, and having grown up with him until now, I have learned to be tolerant and understanding, and no longer to complain. (case1)

In response to social stigmatization, mothers adopt proactive de-stigmatization strategies. They reject the notion that their child’s condition is their fault and instead highlight the shortcomings of social structures and existing systems. Mothers recognize that the social exclusion stems from external forces that fail to provide adequate support and resources, which exacerbates stigma toward both the child and the mother. They also emphasize that parenting is a shared responsibility of the entire family and society, shifting the cultural discourse of responsibility to assert their moral legitimacy. This de-stigmatization challenges traditional views and redefines modern family responsibilities.

## 4 Discussion

This study examined the experiences of mothers raising autistic children and identified several motherhood dilemmas they face, including time imbalance and disrupted parent-child interactions, absence of paternal involvement, unequal parenting responsibilities, as well as stigmatization and inadequate support systems. These challenges impose substantial physical and psychological stress throughout the parenting process. Nevertheless, the mothers demonstrated remarkable resilience. Factors such as adopting fatalistic explanations rooted in traditional medical discourses, internalizing moralized maternal responsibilities, and embracing neurodiverse perspectives contributed to their perseverance. These elements enabled mothers to accept their children’s conditions, persist in caregiving driven by responsibility, and discover hope and meaning in their parenting journey. Beyond caregiving, they developed adaptive strategies such as enhancing personal capacity, negotiating family roles, and reconstructing social support networks to mitigate parenting difficulties, alleviate maternal stress, and balance societal expectations with personal needs. It reflects mothers’ subjective practices within social motherhood’s demands, allowing them to realize self-identity and agency, continuously redefining motherhood through daily life experiences (44).

Our findings align with previous research affirming that raising a child with autism profoundly alters mothers’ life trajectories, introducing significant familial, social, and emotional stressors. Many mothers reported that having an autistic child adversely affected their daily family routines and professional lives, resulting in feelings of marginalization and silence (8). However, rather than being passive victims of stress, mothers actively navigated multiple dilemmas at various life stages (45), seeking joy and meaning in caregiving, motivating value shifts and fostering new life perspectives over time (8). This study contributes to the literature by highlighting the empowering aspects of motherhood for mothers of autistic children, resonating with feminist scholarship on motherhood. Central to maternal empowerment is female autonomy—including control over finances, decision-making authority, and freedom of movement—and resistance against patriarchal oppression (46). As demonstrated in the study, mothers actively adapted their parenting through capacity building, negotiating family responsibilities, reconstructing social support, and empowering themselves within their mothering practices.

It is critical, however, to acknowledge that motherhood can also be a source of oppression, as its idealized demands and normative expectations constrain mothers within dominant discourses (15). At the macro level, our study highlights the insufficiency of China’s social welfare and public service systems to support mothers of autistic children. The Chinese social welfare model emphasizes “increasing family and individual responsibilities while reducing government burdens” (47). Consequently, these mothers primarily rely on personal strengths and social networks for caregiving support. Enhanced care services are urgently needed to empower mothers of children with autism.

## 5 Limitations and strengths

One of the strengths of this study lies in its in-depth exploration of the lived experiences of mothers raising children with autism in the Chinese context. This topic has received limited attention in the existing literature. This study adds new knowledge to the field by highlighting how Chinese cultural narratives, including fatalistic beliefs and moralized maternal roles, shape the way mothers of autistic children interpret and cope with their caregiving responsibilities. Furthermore, the study identifies the strategies mothers employ to reconstruct motherhood under social pressure, contributing to understanding how caregiving can become a site of empowerment. These findings enrich the discourse on autism parenting by introducing a culturally specific and gender-sensitive lens to the experiences of Chinese mothers.

This study also has some limitations. Firstly, among the mothers of children with autism who were interviewed, some had children with severe autism, and some had children with mild autism. Their experiences of motherhood may have differed, which needs to be followed up with in-depth discussions. Secondly, although some mothers discussed aspects of their obstetric history, this was not a formal focus of the research. Future investigations might consider incorporating prenatal and perinatal factors into a more comprehensive inquiry into autism caregiving experiences. Lastly, our study was more qualitative with a small sample size. Thus, the generalization of the findings is limited, and more quantitative studies are needed to compensate for the methodological limitations.

## 6 Conclusion

This study reveals the maternal experiences of mothers raising children with autism within the context of Chinese cultural norms. The findings demonstrate that these mothers face multiple layers of psychological and practical stress during long-term caregiving. At the same time, they exhibit notable resilience and agency in managing these challenges. Rather than being passive recipients of a burden, they actively reconstruct support systems, negotiate family roles, and develop coping strategies that reflect emotional endurance and adaptive strength.

The findings of this study have several important clinical and research implications. Clinically, they highlight the need for more inclusive, family-centered interventions, recognizing mothers’ psychological burdens and caregiving competencies. Mental health professionals should offer culturally sensitive psychosocial support that addresses not only emotional distress but also the empowerment processes and identity transformations experienced by mothers. From a policy perspective, there is an urgent need to strengthen the social support infrastructure for families of children with autism in China. It includes developing accessible respite care services, peer support networks, and flexible employment policies tailored to the needs of caregiving mothers.

## Data Availability

The datasets presented in this article are not publicly available due to privacy and ethical restrictions. The interviews contain sensitive and personally identifiable information about the participants, and public sharing would compromise their confidentiality. Requests to access the datasets should be directed to sduzjun@mail.sdu.edu.cn.
